# The complete mitochondrial genome of *Chaetodon speculum* (Chaetodontiformes, chaetodontidae)

**DOI:** 10.1080/23802359.2021.1906170

**Published:** 2021-03-31

**Authors:** Yukai Yang, Tao Li, Heizhao Lin, Xiaolin Huang, Wei Yu, Zhong Huang

**Affiliations:** a Guangdong Provincial Key Laboratory of Fishery Ecology and Environment, South China Sea Fisheries Research Institute, Chinese Academy of Fishery Sciences, Guangzhou, China;; b Shenzhen Base of South China Sea Fisheries Research Institute, Chinese Academy of Fishery Sciences, Shenzhen, China

**Keywords:** *Chaetodon speculum*: Chaetodontidae: mitochondrial genome

## Abstract

Chaetodontidae species feeding observations showed that they mostly fed on different coral species. Among them, *Chaetodon speculum* (Cuvier, 1831) is one of most important genera of Chaetodontidae, *C. baronessa* and *C. bennetti* appeared to ingest annelid worms during the course of coral feeding, whereas gut contents of *C. punctatofasciatus* and *C. speculum* were dominated by crustaceans. However, the systemically classification and taxonomic studies have so far been limited. In this study, we report the complete mitochondrial genome sequence of *C. speculum*. The mitogenome has 16,537 base pairs (54.4% A + T content) and made up of total of 37 genes (13 protein-coding, 22 transfer RNAs and 2 ribosomal RNAs), and a putative control region. This study will provide useful genetic information for future phylogenetic and taxonomic classification of Chaetodontidae.

The extraordinary diversity of fishes on coral reefs has generated considerable interest in mechanisms that facilitate coexistence of ecologically equivalent species (Pratchett 2005). A member of this biological community, *Chaetodon speculum* which belongs to the Family Chaetodontidae and the Order Chaetodontiformes, is distributed from Indonesia to Japan and south to the Great Barrier Reef and Papua New Guinea, in coastal reef slopes rich with hydroids and anemones. (Nagelkerken et al. 2009).

There is no report of the complete mitogenome of this species *C. speculum*, which was captured in Daya Bay of Shenzhen, Guangdong Province, Republic of China (N22°37′34″, E114°41′06″) in October 2019. Therefore, it is very important to characterize the complete mitogenome of this species, which can be utilized in research on taxonomic resolution, population genetic structure and phylogeography, and phylogenetic relationship. A specimen was deposited at Guangdong Provincial Key Laboratory of Fishery Ecology and Environment, South China Sea Fisheries Research Institute, Chinese Academy of Fishery Sciences (http://feel.scsfri.ac.cn, Dai Ming, daimmy@163.com) under the voucher number CS-1. Total DNA was extracted from muscle following TIANamp Marine Animals DNA Kit (Tiangen, China), The experimental process is performed in accordance with the standard protocol provided by Illumina, including sample quality testing, library construction, library quality testing, library sequencing and other processes and NOVOPlasty software was used to assemble the mitogenomes, the mistake parameter was set by default (Dierckxsens et al. 2017).

In this study, we obtained the complete mitochondrial genome of the *C. speculum*. Its mitochondrial genome has been deposited in the GenBank under accession number MW363870. For a better understanding of genetic status and the evolutionary study, we focused on the genetic information contained in the complete mitochondrial genomes of the fish. The complete mitogenome of the *C. speculum* was 16,537 bp in length, the genomic organization was identical to those of typical vertebrate mitochondrial genomes, including two rRNA genes, 13 protein-coding genes, 22 tRNA genes, a light-strand replication origin (OL), and a putative control region (CR). The overall base composition was 27.8% of A, 26.6% of T, 29.1% of C, and 16.5% of G with a slight A + T bias (54.4%) like other vertebrate mitochondrial genomes. The features mentioned above were accordant with typical Chaetodontidae fish mitogenome.

*Chaetodon speculum* had two non-coding regions, the L-strand replication origin region (36 bp) locating between tRNA-Asn and tRNA-Cys, and the control region (846 bp) locating within the tRNA-Pro and tRNA-Phe. Except for eight tRNA (tRNA-Ser, tRNA-Pro, tRNA-Glu, tRNA-Tyr, tRNA-Cys, tRNA-Asn, tRNA-Ala, and tRNA-Gln) and the *ND6* gene were encoded on the L-strand, the others were encoded on the H-strand. This feature is similar to other fish mitochondrial genes. The complete mitogenome sequence had 16 s RNA (1,664 bp) and 12 s RNA (847 bp), which were located between tRNA-Phe and tRNA-Leu and separated by tRNA-Val gene. The location is same with most vertebrates that have high conservation (Yang et al. 2017).

We built a maximum likelihood tree using the whole mitogenome sequences of *C. speculum* and other 11 closely related species (Figure 1). The phylogeny was constructed based on the General Time Reversible + Invariant + gamma sites (GTR + I + G) model of nucleotide substitution with 1000 bootstrap replicates through RAxML version 8.2.10 (Stamatakis 2014). The results showed that *C. speculum* has the closer relationship with *Chaetodon otofasciatus*.

**Figure 1.**
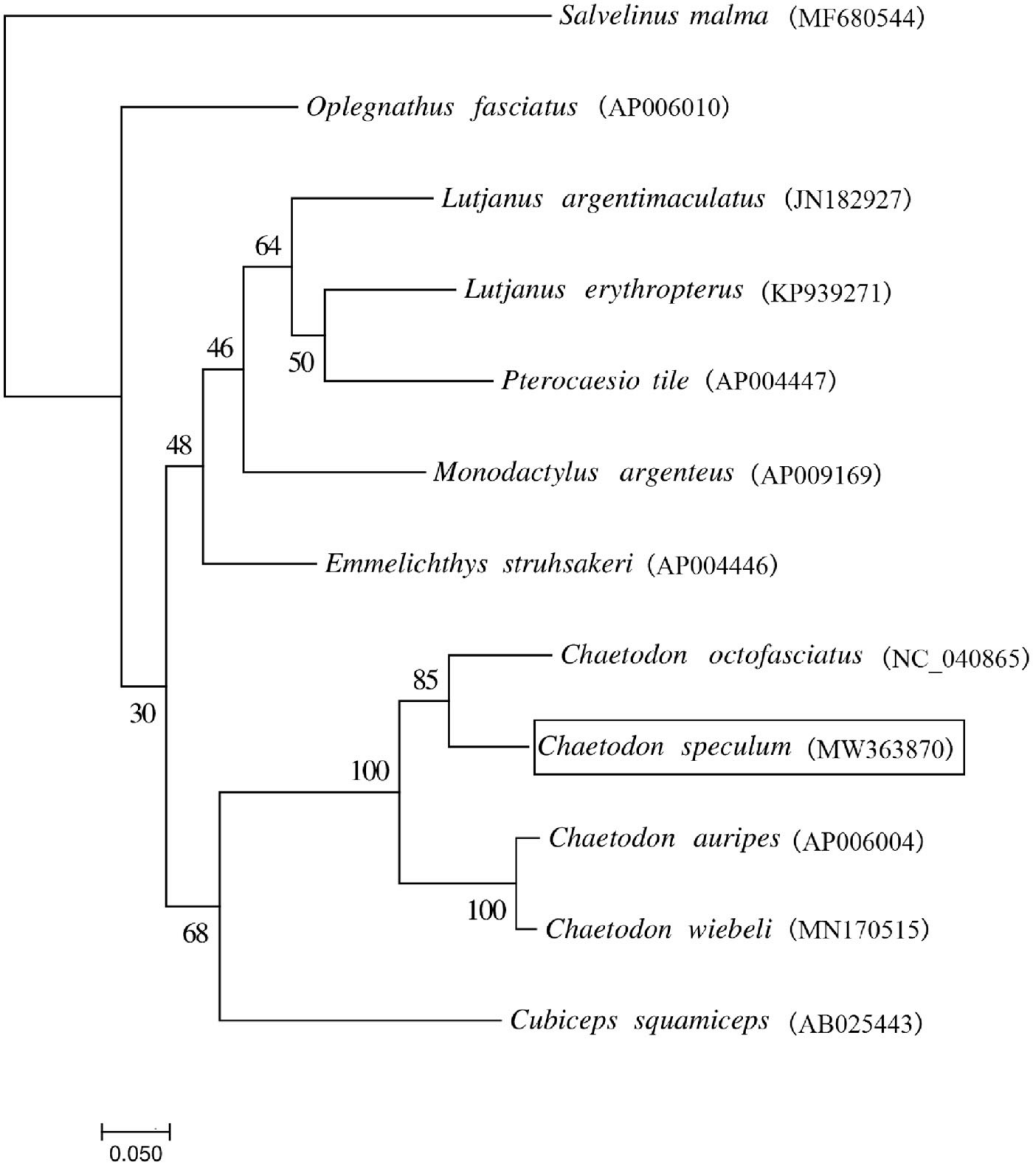
The maximum likelihood tree inferred from the whole mitochondrial genomes of *C. speculum* and other 11 species. The numbers above the branches indicate the bootstrap values.

## Data Availability

The genome sequence data that support the findings of this study are openly available in GenBank of NCBI at https://www.ncbi.nlm.nih.gov/, under the accession no. MW363870.
